# Incidence and influencing factors of 30-day unplanned readmission in chronic heart failure patients: a systematic review and meta-analysis

**DOI:** 10.3389/fcvm.2025.1663018

**Published:** 2026-01-14

**Authors:** Tongtong Chen, Renxiu Wang, Hongxia Song

**Affiliations:** Department of Nursing, The First Affiliated Hospital of Shandong First Medical University & Shandong Provincial Qianfoshan Hospital, Jinan, China

**Keywords:** chronic heart failure, readmission, incidence, risk factors, systematic review, meta-analysis

## Abstract

**Objective:**

Systematic review and meta-analysis of the incidence and risk factors for 30-day unplanned readmissions in patients with chronic heart failure(CHF).

**Methods:**

We searched PubMed, Embase, Web of Science, Scopus, Medline, CINAHL, and Chinese databases up to February 2025. Data were analyzed by using Stata 17.0.

**Results:**

Among 4,040 screened publications, 21 studies were included. The incidence of 30-day unplanned readmission in CHF patients was 17.7% (95% CI: 13.9%–21.5%). Age ≥65 years (OR = 1.35, *P* = 0.024), diagnosed with chronic kidney disease (CKD) (OR = 1.26, *P* = 0.000), diabetes (OR = 1.49, *P* = 0.001), atrial fibrillation (AF) (OR = 1.12, *P* = 0.005), coronary heart disease (CHD) (OR = 5.28, *P* = 0.000), cardiomyopathy (OR = 1.44, *P* = 0.000), NYHA class ≥Ⅲ or Ⅳ (OR = 1.64, *P* = 0.000), use of beta blockers (OR = 1.25, *P* = 0.000), loop diuretics (OR = 1.41, *P* = 0.004), thiazides (OR = 1.22, *P* = 0.000), LVEF < 40% (OR = 1.44, *P* = 0.000), and length of stay (LOS) (OR = 1.16, *P* = 0.000) were risk factors for 30-day unplanned readmission in CHF patients.

**Conclusions:**

The incidence of 30-day unplanned readmissions in patients with CHF is moderate but concerning. Accurate identification of identified risk factors for targeted interventions to reduce the need for readmissions.

**Systematic Review Registration:**

https://www.crd.york.ac.uk/PROSPERO/view/CRD42024610843, PROSPERO CRD42024610843.

## 1 Introduction

Chronic heart failure (CHF) is the end stage of all types of cardiovascular diseases, and its incidence is rising globally (1). Current estimates indicate that approximately 64.3 million individuals worldwide are affected by HF, resulting in treatment costs as high as $100 billion annually. Notably, over 60% of these expenditures are attributed to managing CHF patients who require readmission within one year of discharge (2). Moreover, nearly one-third of these readmissions occur unexpectedly within just 30 days post-discharge, highlighting the substantial clinical and economic burden associated with this condition (3).

Unplanned readmissions, which are defined as unanticipated readmissions within 30 days of discharge for the same or a related condition, are a significant indicator of healthcare quality and are strongly associated with the standard of medical care and discharge planning during the patient's most recent hospitalization (4). Research indicates that up to 25% of unplanned readmissions for CHF could be preventable (5).

Several studies have examined the incidence of 30-day unplanned readmission among CHF patients (6); however, there are significant differences in the findings due to factors such as the study population, geographic region, and sample size. To date, no comprehensive systematic review has been conducted on both the incidence rates and associated risk factors for 30-day unplanned readmissions in this patient group. Thus, this study quantitatively synthesizes the available evidence to identify predictors influencing 30-day unplanned readmission in CHF patients. The findings will facilitate early risk identification, preventive interventions, and optimized nursing management, ultimately improving patient outcomes while reducing the economic burden on healthcare systems.

## 2 Methods

This systematic review and meta-analysis was conducted according to the Preferred Reporting Items for Systematic Review and Meta-Analyses (PRISMA) guidelines. It was registered in the National Institute for Health Research PROSPERO database with registration number CRD42024610843.

### 2.1 Search strategy

We searched PubMed, Web of Science, Embase, CINAHL, Medline, Scopus, China Knowledge Network (CNKI), Wanfang Database, Chinese Biomedical Database (CBM), and Chinese Science and Technology Journal Database (VIP) from inception to February 28, 2025. The search strategies used a combination of MeSH terms and entry terms. The search terms included heart failure, cardiac failure, heart decompensation, patient readmission*, unplanned readmission*, 30-day readmission*, hospital readmission*, risk factor*, influencing factor*, impact factor*, dangerous factor*, influence factor*, predictive factor*, contributing factor*, relevant factor*, relevant factor*, correlative factor*, associated factor*, prevalence*, incidence. The detailed search strategies are shown in Supplementary Appendix A1.

### 2.2 Study inclusion and exclusion criteria

The inclusion criteria were as follows: (1) Study population: patients diagnosed with chronic heart failure (CHF) and aged ≥18 years; (2) Type of study: observational studies, including cohort studies, case-control studies and cross-sectional studies; (3) Study content: the study focuses on unplanned readmissions within 30 days post-discharge among patients with CHF; (4) Outcome indicator: incidence and/or risk factors of 30-day unplanned readmissions.

The exclusion criteria were as follows: (1) Literature with incomplete information or from which the full text cannot be obtained; (2) Duplicate publication; (3) Non-Chinese and English literature.

### 2.3 Study selection

Two researchers with training in evidence-based research independently screened, retrieved, and cross-checked the literature. Any disagreements were resolved through discussion with a third researcher to reach a consensus. Duplicated literature was excluded by using the literature management program Endnote X9. The titles and abstracts were initially read to eliminate any irrelevant literature, and then the full texts were read for re-screening to determine the final literature to be included.

### 2.4 Data extraction

Two reviewers independently reviewed and extracted data from the included studies. They extracted relevant information from the last included studies using a standardized data extraction form, including first author, publication year, region, study design, sample size, the incidence of 30-day unplanned readmissions in CHF, influencing factors, and Odds ratio (OR) or Hazard ratio (HR) with their corresponding 95% confidence intervals (CIs).

### 2.5 Quality assessment

Two researchers independently assessed the quality of the included literature using a back-to-back approach, applying the most appropriate quality assessment tool based on the type of literature. In cases of conflicting opinions, a third researcher was consulted to resolve discrepancies. The methodological quality of cohort and case-control studies was assessed using the Newcastle-Ottawa Quality Assessment Scale (NOS) (7).

Three aspects make up the NOS: selection, comparability, and exposure. Each dimension is evaluated using 8 items for a total score of 9 points. The quality of cross-sectional studies was evaluated according to the quality evaluation criteria for cross-sectional studies recommended by the Agency for Health Care Research and Quality (AHRQ) (8), which contains 11 entries, with a total score of 11 points. A rating of 0–3 indicates low quality, 4–7 indicates medium quality, and 8–11 indicates high quality.

### 2.6 Statistical analysis

In this study, a meta-analysis of the incidence and risk factors of 30-day unplanned readmissions in CHF patients was performed using Stata 17.0 software. The OR and 95% CI were used to express the count data, and the MD and 95% CI were selected for the measurement data. Moreover, the I^2^ was used to evaluate the statistical heterogeneity. In addition, a meta-analysis was performed by using a random effects model when *P* < 0.1 and/or *I*^2^ > 50%. When *P* ≥ 0.1 and *I*^2^ ≤ 50%, the data were retested by using a fix effects model. Egger linear regression tests and funnel plots were combined to assess potential publication bias, and *P* > 0.05 suggested that there was no publication bias.

## 3 Results

### 3.1 Search results

**Figure 1:**
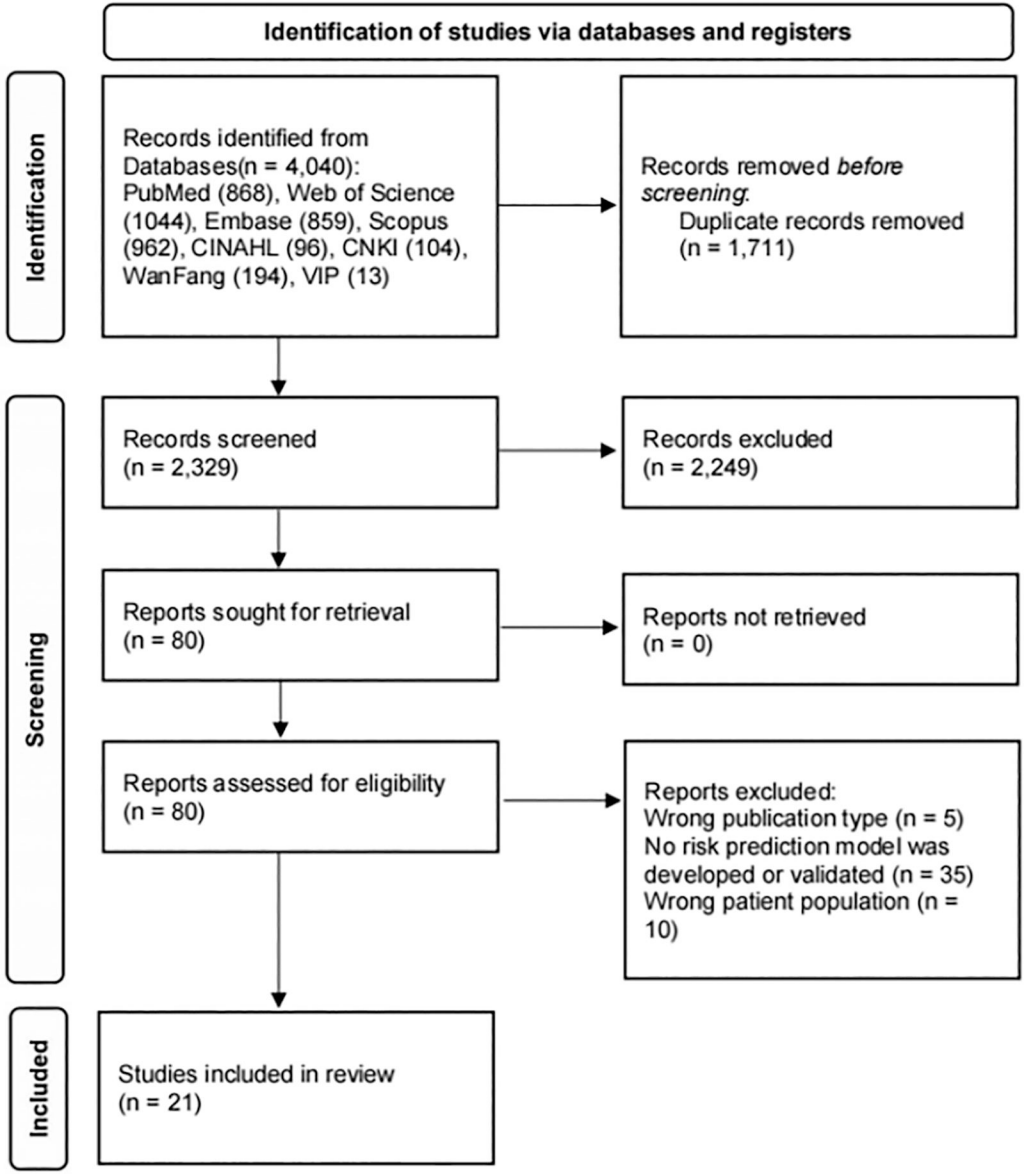
Flowchart of study inclusion.

The initial literature search identified 4,040 studies, which were reduced to 2,329 after the removal of duplicates. After the screening of titles and abstracts, 80 articles were retrieved for full-text evaluation. Ultimately, 21 studies (9–29) met the inclusion criteria and were incorporated into this meta-analysis. The study flowchart according to the PRISMA statement is shown in Figure 1.

### 3.2 Study characteristics

The sixteen included studies were mainly from the USA (*n* = 10) (9–18), China (*n* = 4) (19–22), Japan (*n* = 2) (23, 24), Korea (*n* = 2) (25, 26), Lebanon (*n* = 1) (27), Canada (*n* = 1) (28), and Ethiopia (*n* = 1) (29). The 21 studies comprised 18 cohort studies, two case-control studies, and one cross-sectional study. Among them, there were 18 English papers and 3 Chinese papers. The sample size (excluding missing data) ranged from 187 to 10,978,056. The predicted results were 30-day unplanned readmission, as shown in Table 1.

**Table 1:** Characteristics of included studies.

ID	Study	Country	Study type	Sample size	Male (%)	CHF (n)	Overall incidence	Risk factors included	Quality score	
									NOS	AHRQ
1	Alzaghari 2019 (9)	US	Cross-sectional study	270	140 (52)	138	51	CKD, use of CPAP machine, BNP		7
2	Arora 2016 (10)	US	Retrospective study	301,892	(49.4)	64,264	21.29	CCI, obesity, DM, CPD, peripheral vascular disease, renal failure, electrolyte imbalance, anemia, coagulation defect, depression, psychosis, substance abuse, index admission type(non elective/elective), disposition(home/facility/others), LOS	8	
3	Carlson 2019 (11)	US	Retrospective study	189	79 (42)	10	5.3	emergent admission, AF	6	
4	Fudim 2017 (12)	US	Retrospective study	6,584	(66)	751	11.00	chronic respiratory disease, cerebrovascular disease, cardiac resynchronization therapy, loop diuretics, ACEI or ARB	8	
5	Kwok 2019 (13)	US	Retrospective study	109,780	52,936 (48.22)	16,576	15.1	renal failure, cancer, circulatory support, discharge against medical advice	7	
6	Mirkin 2017 (14)	US	Retrospective study	155,146	74,082 (47.75)	35,294	22.8	sex, race, coverage by medicare, comorbidity, discharge to a skilled nursing facility or with a home nurse, LOS, admission from another facility, emergent admission	8	
7	Gusto 2023 (15)	US	Case-control study	450	234 (52)	–	–	Living arrangements, CRF		7
8	Jain 2023 (16)	US	Retrospective study	48,971	26,542	10,370	21.2	sex, socioeconomic status, index admission type(non elective/elective), AF, COPD, CKD, anemia, CCI	6	
9	Jha 2022 (17)	US	Retrospective study	60,514	23,358	12,708	21	medicaid insurance, Charlson co-morbidity score, LOS, age, sex	6	
10	Kim 2023 (18)	US	Retrospective study	43,111	15,804	8,794	20.4	Age, Elixhauser co-morbidity indexes, number of diagnoses, renal failure	6	
11	Shao 2021 (19)	China	Retrospective study	3,129	1,473 (47.08)	713	22.79	DM, renal insufficiency, AMI, LOS, quality of post-discharge care	7	
12	Hu 2022 (20)	China	Prospective study	2,254	1,324 (58.74)	101	7.1	LVEF, left ventricular end-diastolic size, hs-cTn, NT-proBNP, hemoglobin, RDW-CV, albumin	9	
13	Zhang 2023 (21)	China	Retrospective study	14,843	6,713 (45.227)	1,479	9.964	NT-proBNP, LOS, triglycerides, blood phosphorus, blood potassium, lactate dehydrogenase	8	
14	Zhuang 2022 (22)	China	Retrospective study	390	239 (61.28)	–	–	alcohol abuse, regular exercise, cardiac function classification, hypertension, DM, CHD, hyperlipidemia, arrhythmia	6	
15	Aizawa 2015 (23)	Japan	Case-control study	68,257	2,351 (52.5)	4,479	6.56	age, NYHA classification, CCI, beta-blockers, loop diuretics, thiazide, nitrates, LOS, BMI, ACEI or ARB, CCB, spironolactone	7	
16	Miyazaki 2023 (24)	Japan	Retrospective study	180,125	93,182	10,283	5.71	LOS,sex, smoking, age, BMI, barthel index, artificial ventilator, beta-blockers, thiazides, tolvaptan, loop diuretics, carperitide, class Ⅲ antiarrhythmic agents, MI, DM, renal disease, AF, dilated cardiomyopathy, discharge to home	7	
17	Chung 2017 (25)	Korea	Retrospective study	19,128	8,918 (46.6)	5,286	27.6	paralysis, metastatic cancer, loop diuretics, ACEI or ARB	9	
18	Lim 2018 (26)	Korea	Prospective study	4,566	2,347 (51.40)	446	9.8	age, NYHA classification, hypertension, HF, COPD, etiology of cardiomyopathy, SBP, LVEF, serum sodium level, BNP or NT-proBNP, beta blockers, ACEI or ARB	8	
19	Deeka 2016 (27)	Lebanon	Retrospective study	187	111 (59.40)	28	15.00	DM, CAD, LOS, gamma glutamyl transpeptidase levels	7	
20	Sharma 2022 (28)	Canada	Prospective study	9,845	5,539 (56)	2,165	22.00	–	9	
21	Ayenew 2024 (29)	Ethiopia	Retrospective study	572	303 (52.8)	151	26.4	age, rural residency, comorbidity, platelet, volume, hemoglobin	9	

ACEI, angiotensin converting enzyme inhibitors; AF, atrial fibrillation; ARB, angiotensin II receptor blockers; AMI, acute myocardial infarction; BMI, Body mass index; BNP, B-type Natriuretic Peptide; CAD, coronary artery disease; CCB, calcium channel blockers; CCI, Charlson Comorbidity Index; CHD, coronary heart disease; CKD, chronic kidney disease; COPD, chronic obstructive pulmonary disease; CPAP, continuous positive airway pressure; CPD, chronic pulmonary disease; CRF, chronic renal failure; DM, diabetes mellitus; hs-cTn, hypersensitive cardiac troponin T; LOS, length of stay; LVEF, left ventricular ejection fraction; MI, myocardial infarction; NT-proBNP, N-terminal pro-brain natriuretic peptide; NYHA, New York Heart Association; RDW-CV, coefficient of variation of red cell distribution width; SBP, systolic blood pressure.

### 3.3 Quality assessment

Among the included studies, one cross-sectional study (9) received a quality score of 7, two case-control studies (15, 23) each scored 7 points, and eighteen cohort studies (10–14, 16–22, 24–29) received scores ranging from 6 to 9. Overall, the results of the literature quality evaluation showed that nine of the 21 literature were of high quality, and 12 were of medium quality.

### 3.4 The overall incidence of 30-day unplanned readmission in CHF patients

**Figure 2:**
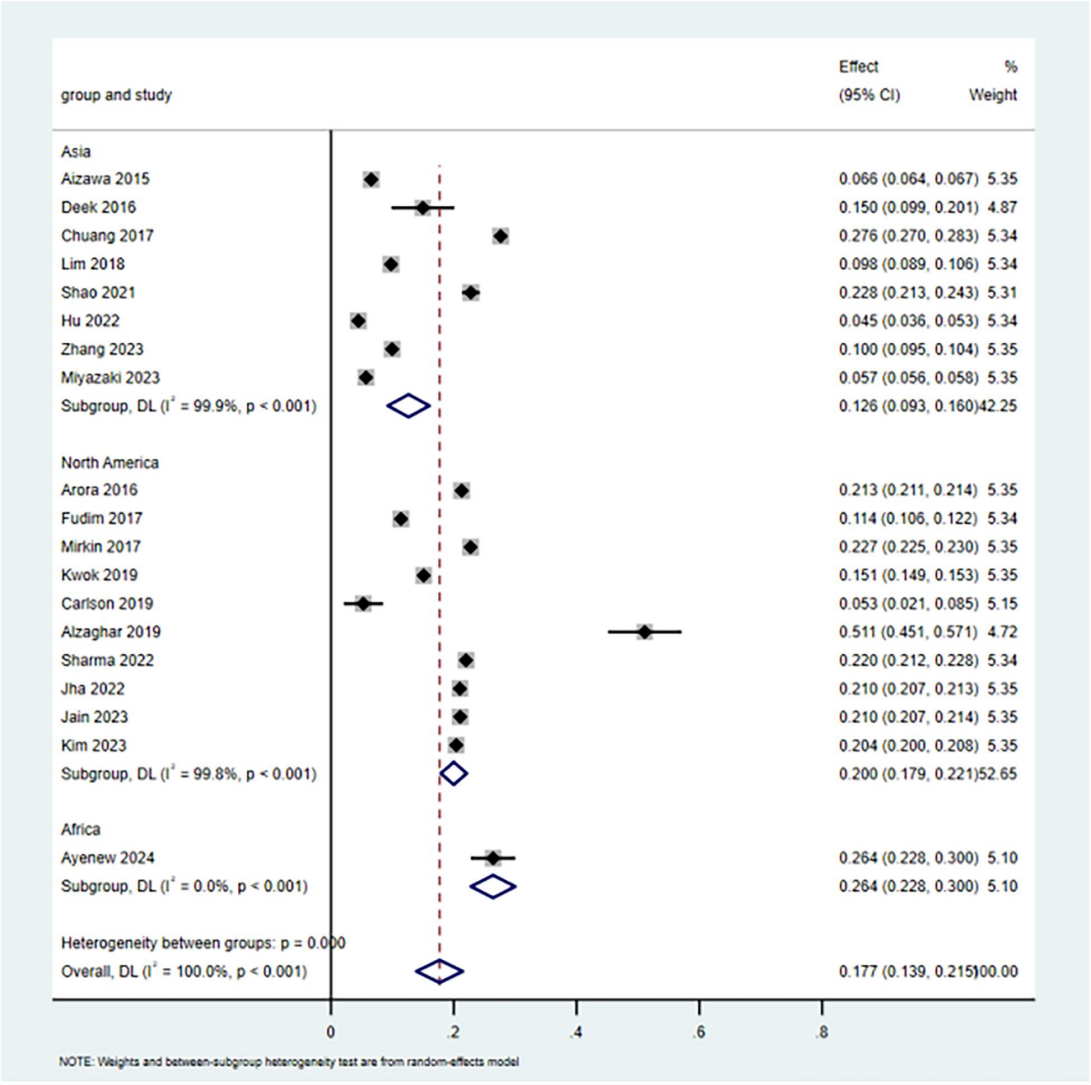
The incidence of 30-day unplanned readmission in CHF patients.

Of the 21 included studies, nineteen studies (9–14, 16–21, 23–29) reported the incidence of 30-day unplanned readmissions. Of the 19 included studies available for meta-analysis, the incidence of 30-day unplanned readmission ranged from 6% to 51%. As there was high heterogeneity between studies [*I*^2^ = 99.966%, *P* < 0.001], a random-effects model was employed for the meta-analysis. The estimated incidence of 30-day unplanned readmission was 17.7% (95% CI: 13.9%–21.5%). Subgroup analysis by region revealed the incidence of 20.2% (95% CI: 17.9%–22.1%) in North America, 12.6% (95% CI: 9.3%–16.0%) in Asia, and 26.4% (95% CI: 22.8%–30.2%) in Africa. The results of the study were relatively strong, and sensitivity analysis showed that the overall incidence did not change significantly when individual studies were excluded on a case-by-case basis (Figure 2).

### 3.5 Meta-analysis results

A total of 83 risk factors were included in the study, of which 27 risk factors were eligible for meta-analysis: age, sex, NYHA classification, the longer length of stay(LOS), angiotensin-converting enzyme inhibitors (ACEI) or angiotensin II receptor blockers(ARB), chronic obstructive pulmonary disease(COPD), chronic kidney disease(CKD), beta-blockers, renal failure, loop diuretics, thiazide, DM, LVEF, BNP or NT-proBNP, body mass index(BMI), index admission type(non elective/elective), coronary heart disease(CHD), acute myocardial infarction(AMI),medicaid insurance, hemoglobin, atrial fibrillation(AF), complications, use of artificial ventilator, hypertension, and cardiomyopathy. These 24 risk factors can be divided into four categories: demographic factors, disease-related factors, drug-related factors, and hospitalization-related factors, as shown in Table 2, the forest plots of risk factors for 30-day unplanned readmissions in patients with CHF are shown in Supplementary Appendix A2.

**Table 2:** Meta-analysis results of risk factors for 30-day unplanned readmission in patients with CHF.

Risk factors	Number of studies	Heterogeneity test		Effects model	Meta-analysis results	
		*I*^2^ (%)	*P*		Odds ration(OR)	*P*
Demographic factors						
Age ≥65	5	99.6	<0.001	Random	1.35 (1.04,1.75)	0.024
Female	4	95.5	<0.001	Random	1.03 (0.92,1.15)	0.625
Medicaid insurance	2	97.8	<.001	Random	0.96 (0.67,1.37)	0.807
Disease-related factors						
COPD	2	70.9	0.064	Random	1.28 (1.00,1.64)	0.054
CKD	7	72.9	0.001	Random	1.26 (1.21,1.32)	0.000
DM	5	87.9	<0.001	Random	1.49 (1.17,1.89)	0.001
AF	3	69.2	0.039	Random	1.12 (1.04–1.22)	0.005
CHD	2	63.8	0.096	Random	5.28 (2.52,11.04)	0.000
Complication	2	76.6	0.039	Random	1.27 (0.87,1.85)	0.216
AMI	2	78.9	0.03	Random	1.61 (0.77,3.40)	0.209
Hypertension	2	85.4	0.009	Random	1.96 (0.85,4.50)	0.113
Cardiomyopathy	2	0	0.804	Fix	1.44 (1.26,1.65)	0.000
NYHA class ≥Ⅲ or Ⅳ	2	0	0.767	Fix	1.64 (1.44,1.86)	0.000
Use of artificial ventilator	2	83.4	0.014	Random	0.64 (0.16,2.58)	0.533
Drug factors						
ACEI or ARB	4	93.1	<0.001	Random	0.88 (0.72,1.08)	0.216
Beta-blockers	3	60.5	0.08	Random	1.25 (1.12,1.38)	0.000
Loop diuretics	3	84.8	0.001	Random	1.41 (1.12,1.78)	0.004
Thiazide	2	21.9	0.258	Fix	1.22 (1.14,1.30)	0.000
Laboratory indications						
LVEF < 40%	2	0	0.497	Fix	1.44 (1.19,1.75)	0.000
BNP ≥ 400 pg/mL or NTproBNP ≥ 1,800 pg/mL	4	93.3	<0.001	Random	1.53 (0.95,2.44)	0.078
Hemoglobin < 110 g/L	2	94.7	<0.001	Random	2.97 (0.71,12.40)	0.136
BMI	2	97.5	<0.001	Random	0.80 (0.54,1.18)	0.263
Hospitalization-related factors						
Non-elective Admission(emergent admission)	4	98.8	<0.001	Random	1.15 (0.89,1.48)	0.297
LOS	8	98.9	<0.001	Random	1.16(1.08,1.24)	0.000

ACEI, angiotensin converting enzyme inhibitors; AF, atrial fibrillation; ARB, angiotensin II receptor blockers; AMI, acute myocardial infarction; BMI, Body mass index; BNP, B-type Natriuretic Peptide; CHD, coronary heart disease; CKD, chronic kidney disease; COPD, chronic obstructive pulmonary disease; DM, diabetes mellitus; LOS, length of stay; LVEF, left ventricular ejection fraction; MI,myocardial infarction; NT-proBNP, N-terminal pro-brain natriuretic peptide; NYHA, New York Heart Association.

#### 3.5.1 Demographic factors

The meta-analysis showed that age ≥65 years [OR = 1.35, 95% CI (1.04, 1.75), *P* = 0.024] was a risk factor for 30-day unplanned readmissions in patients with CHF. Moreover, female sex [OR = 1.03, 95% CI (0.92, 1.15), *P* = 0.625] and Medicaid insurance coverage [OR = 0.96, 95% CI (0.67, 1.37), *P* = 0.807] were not associated with the development of 30-day unplanned readmission in patients with CHF.

#### 3.5.2 Disease-related factors

The meta-analysis showed that diagnosed with CKD[OR = 1.26, 95%CI (1.21,1.32), *P* = 0.000], diabetes[OR = 1.49, 95%CI (1.17,1.89), *P* = 0.001], AF[OR = 1.12, 95%CI (1.04,1.22), *P* = 0.005], CHD[OR = 5.28, 95%CI (2.52,11.40), *P* = 0.000], cardiomyopathy[OR = 1.44, 95%CI (1.26,1.65), *P* = 0.000], and NYHA class ≥Ⅲ or Ⅳ[OR = 1.64, 95%CI (1.44,1.86), *P* = 0.000], were risk factors for 30-day unplanned readmission in patients with CHF. However, diagnosed with COPD [OR = 1.28, 95%CI (1.10,1.64), *P* = 0.054], AMI[OR = 1.61, 95%CI (0.77,3.40), *P* = 0.209], hypertension[OR = 1.96, 95%CI (0.85,4.50), *P* = 0.113], with complication(OR = 1.27, and use of artificial ventilator[OR = 0.64, 95%CI (0.16,2.58), *P* = 0.533],95%CI (0.87,1.85), *P* = 0.216), were not associated with the development of 30-day unplanned readmission in patients with CHF.

#### 3.5.3 Drug factors

The meta-analysis showed that the use of beta blockers at discharge [OR = 1.25, 95% CI (1.12, 1.38), *P* = 0.000], loop diuretics [OR = 1.41, 95% CI (1.12, 1.78), *P* = 0.004], and thiazides [OR = 1.22, 95% CI (1.14, 1.30), *P* = 0.000] were risk factors for 30-day unplanned readmission in patients with CHF. Moreover, the prescription of ACEI or ARB at discharge [OR = 0.88, 95%CI (0.16,2.58), *P* = 0.216] was not associated with the development of 30-day unplanned readmission in patients with CHF.

#### 3.5.4 Laboratory indications

The meta-analysis showed that LVEF < 40%[OR = 1.44, 95%CI(1.19,1.75), *P* = 0.000] was a risk factor for 30-day unplanned readmission in patients with CHF. However, BNP ≥ 400 pg/mL or NTproBNP ≥ 1,800 pg/mL[OR = 1.53, 95%CI(0.95,2.44), *P* = 0.078], haemoglobin <110 g/L[OR = 2.97, 95%CI(0.71,12.40), *P* = 0.136], and BMI ≥ 30 kg/m^2^[OR = 0.80, 95%CI(0.54,1.18), *P* = 0.297] were not associated with the development of 30-day unplanned readmission in patients with CHF.

#### 3.5.5 Hospitalization-related factors

The meta-analysis showed that longer LOS in the hospital[OR = 1.16, 95%CI(1.08,1.24), *P* = 0.000] was a risk factor for 30-day unplanned readmission in patients with CHF. Moreover, non-elective admission(emergent admission)[OR = 1.15, 95%CI(0.89,1.48), *P* = 0.297] was not associated with the development of 30-day unplanned readmission in patients with CHF.

### 3.6 Meta-analyses heterogeneity and publication bias

#### 3.6.1 Sensitivity analyses

**Figure 3:**
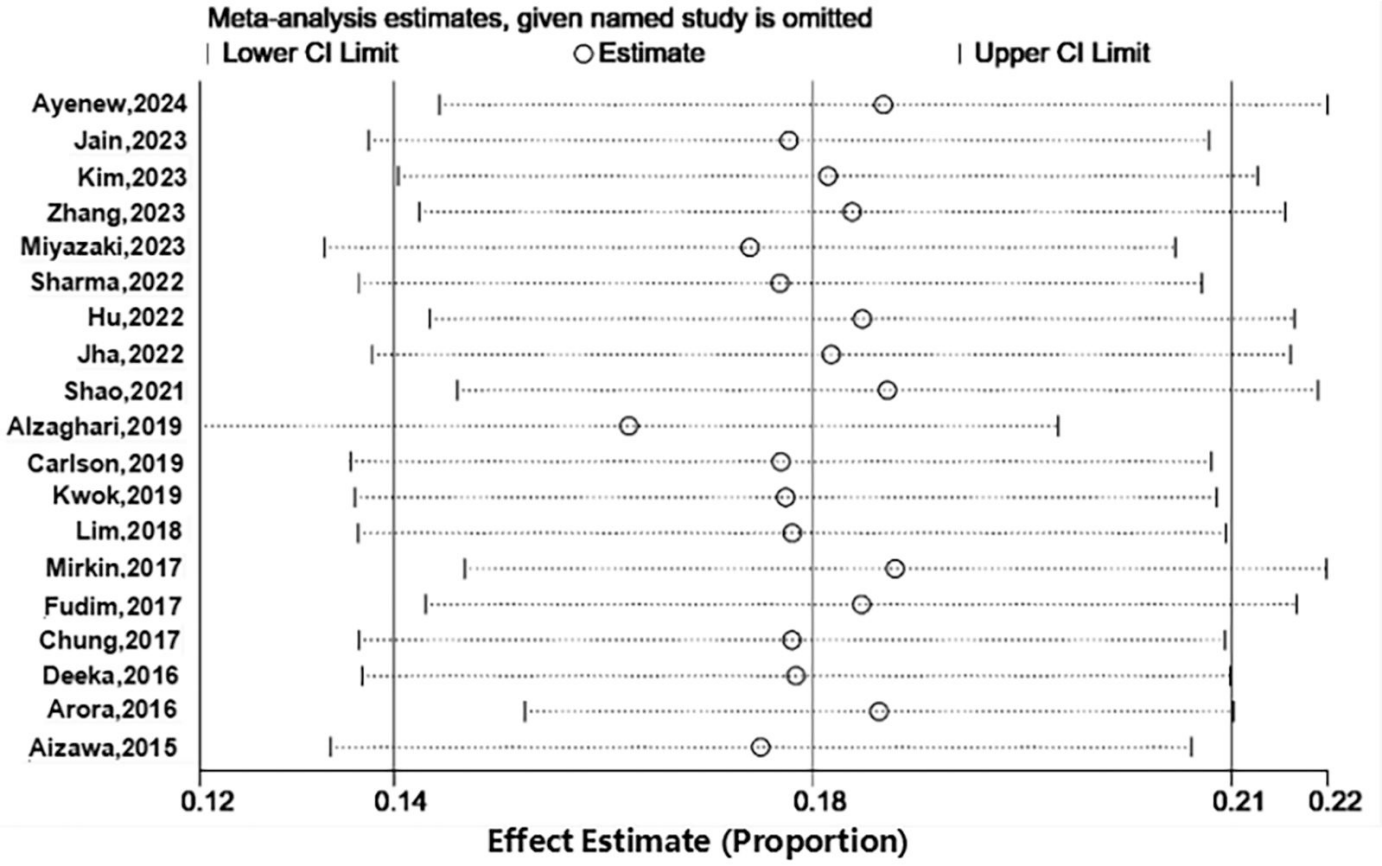
Sensitive analysis of prevalence.

We performed a sensitivity analysis to furtherly explore the potential origin of heterogeneity by detaching each article from our meta-analysis independently. As shown in Figure 3, the pooled incidence demonstrated that our results were credible, and omitting any article did not obviously affect study heterogeneity.

#### 3.6.2 Publication bias

**Figure 4:**
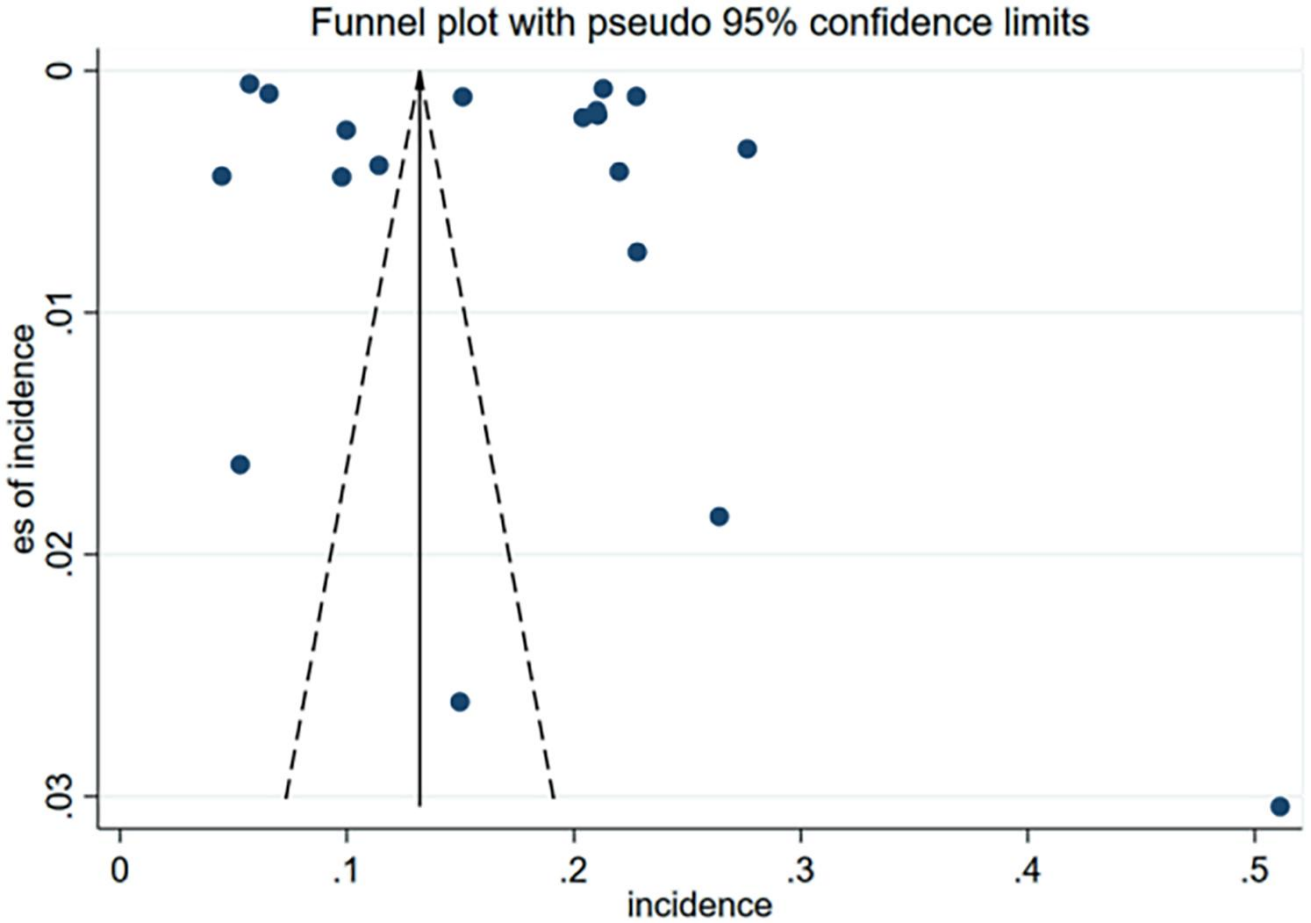
Funnel plot of incidence for publication bias of included studies.

**Figure 5:**
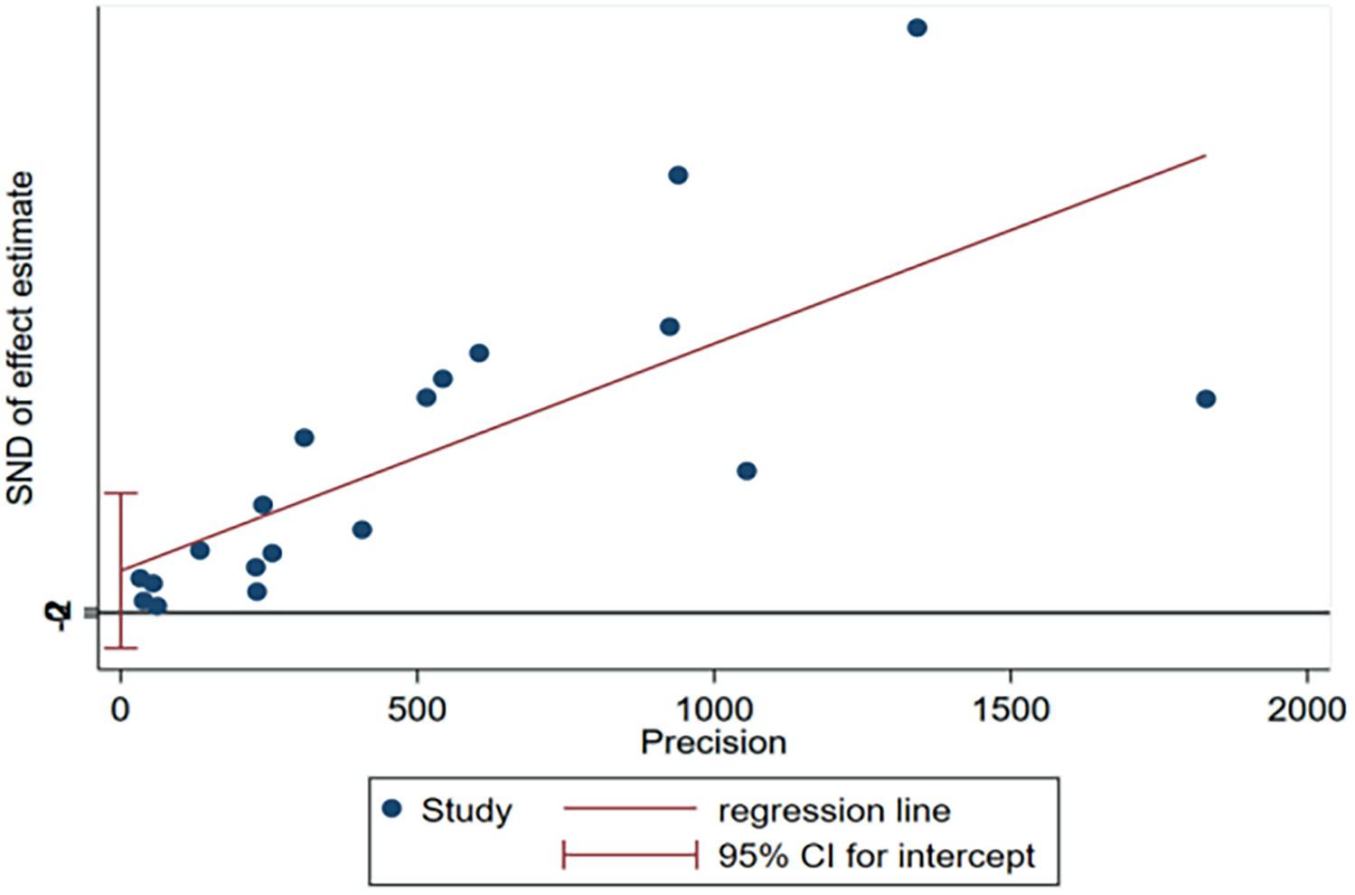
Egger's test plot of incidence for publication bias of included studies.

No significant publication bias was observed for the incidence of 30-day unplanned readmission in the funnel plot (Figure 4) or result from Egger test (Figure 5). As the number of articles analyzed on each risk factor was less than 10, we did not further analyze the publication bias.

## 4 Discussion

### 4.1 The incidence of 30-day unplanned readmission for CHF patients is moderate, but still a cause for concern

The results of this study demonstrated a 17.7% (95% CI: 13.9%–21.5%) incidence of 30-day unplanned readmission for CHF patients, which is consistent with the findings of previous studies. However, the meta-analysis revealed substantial heterogeneity, potentially attributable to variations in study sample sizes, diverse data sources, and differences in heart failure subtypes across the included studies (30). Subgroup analysis revealed regional variations in the incidence of 30-day unplanned readmissions among patients with CHF. Africa demonstrated the highest incidence, followed by North America, while Asia showed the lowest rates. These geographical disparities can be explained by differences in healthcare infrastructure, socioeconomic conditions, and post-discharge care systems. In many African countries, economic instability,limited hospital capacity, shortages of trained personnel, and uneven distribution of resources make structured follow-up difficult and raise the likelihood of readmission (29). In contrast, North America benefits from advanced medical technologies and specialized heart failure programs, yet care coordination remains inconsistent (31). Fragmented systems, variable insurance coverage, and gaps in follow-up planning often leave patients vulnerable to deterioration once they leave the hospital (32). Some high-income Asian nations present another picture: well-organized primary care networks and strong hospital–community links may facilitate smoother transitions, though inequities persist in rural and low-income areas. Meanwhile, emerging strategies in Asia, including government-funded home visits, community health worker involvement, and integrated electronic health records—suggest promising avenues for strengthening continuity of care (33). Moreover, a significant methodological issue is to whether CHF was recognized as the primary cause of hospitalization or as a secondary diagnosis alongside other acute conditions. Numerous studies failed to distinctly differentiate among these diagnostic groups (34). For example, clinical practice reveals that while some chronic patients are admitted with other symptoms as the primary diagnosis, these symptoms are often triggered by heart failure. Therefore, it is challenging to strictly distinguish whether patients are admitted with CHF as the primary or secondary diagnosis (35). This discrepancy may have resulted in case-mix heterogeneity. Including both diagnostic groups indiscriminately may consequently either inflate or deflate the actual incidence of CHF readmissions (36). This meta analysis notably did not find any pertinent studies from Europe; nonetheless, of the four principal papers discovered regarding post-discharge “home” BNP/NT-proBNP follow-up, three originated from the UK (37–39). This presents a paradox: although European researchers have significantly contributed to assessing the feasibility and potential advantages of home BNP monitoring, they have not supplied analogous intra-hospital datasets for indicators predicting early readmission. In contrast, researchers from several regions, including North America, Asia, and Africa, have amassed substantial in-hospital prognostic data but have contributed minimally to the evidence base for home BNP monitoring post-discharge. The disparities in location indicate an imbalance in the global research landscape regarding CHF management. Some areas focus more on inpatient risk profiling, while others focus more on outpatient biomarker-guided care. To fill in these gaps, countries will need to work together to pay attention to the incidence of readmission, find in-hospital predictors and evaluate structured post-discharge interventions. This will make sure that there is more complete and regionally representative evidence to guide practice (40).

### 4.2 Risk factors for 30-day unplanned readmission in patients with CHF are high and should be better identified

#### 4.2.1 Demographic factors

The results of meta-analysis showed that age ≥65 years was a risk factor for the 30-day unplanned readmission in patients with CHF. In 2020, a study showed that HF was one of the most common diagnoses in hospitalized older patients aged >65 years (41). According to the Report on Cardiovascular Health and Diseases in China: An Updated Summary of 2023, the risk of HF prevalence increases with age, 3.09% in people aged 60–79 years and 7.55% in people over 80 years (42). In 2015, a study showed that the incidence of HF was significantly higher in the elderly than in the young and middle-aged, with the risk of incidence increasing approximately 1-fold for every 10-year increase in age (43). However, the age dispersion of study populations may also lead to potential selection bias. Inconsistent reporting of age-stratified analyses across studies impeded the assessment of age-specific risk gradients for readmission. Older persons often exhibit heightened frailty, cognitive impairments, and functional decline, which impact post-discharge self-management and compliance. In the absence of appropriate age-stratified reporting, aggregated estimates may obscure clinically important disparities in risk profiles between younger and older geriatric patients.

#### 4.2.2 Disease-related factors

This study found that CKD, DM, AF, CHD, NYHA, and cardiomyopathy were risk factors for the 30-day unplanned readmission in patients with CHF. Individuals with ischemic CHF, frequently attributed to CAD, typically experience concurrent health issues, including DM, CKD, and peripheral vascular disease (PVD). The presence of these comorbidities, coupled with the risk of recurrent ischemia or arrhythmia, may increase the likelihood of rapid deterioration in the patient's condition, necessitating hospital readmission (44). Conversely, non-ischemic etiologies such as cardiomyopathy may have a distinct trajectory, with decompensation frequently associated with increasing pump failure, arrhythmias, or uncontrolled hypertension. Numerous observational studies have demonstrated that ischemia etiologies are associated with elevated short-term readmission and death rates compared to non-ischemic causes (44). The strength of this association may be contingent upon the adherence of medical care to established recommendations. From a clinical standpoint, distinguishing between ischemia and non-ischemic congestive heart failure is essential for risk evaluation and determining follow-up intensity. Ischemic CHF patients may benefit from enhanced post-hospitalization monitoring, secondary prevention programs targeting coronary disease, and early cardiac rehabilitation. Conversely, the care of non-ischemic instances may concentrate on regulating the underlying conditions that precipitated the issue (45). Moreover, the distribution and impact of comorbidities constitute a considerable source of potential bias. The majority of individuals with CHF, particularly the elderly, possess many chronic health conditions. These comorbidities influence both the probability of initial hospitalization and the risk of subsequent early readmission (46). Nevertheless, the studies analyzed demonstrated considerable diversity in their definitions, measures, and adjustments for comorbidities, leading to residual confounding. In other cases, comorbidities were recorded only after discharge or obtained from administrative databases, which may result in an underrepresentation of their prevalence and reduce the comparability of findings between studies.

#### 4.2.3 Drug factors

The results of the meta-analysis showed that the use of beta-blockers, loop diuretics, and thiazides at discharge was a risk factor for 30-day unplanned readmission in patients with CHF. Beta-blockers are important for reducing long-term mortality and hospitalization rates. Nevertheless, Aizawa et al. (23) claim that initiating or escalating the dosage may temporarily exacerbate symptoms due to their negative inotropic and chronotropic effects, particularly in those with advanced disease. This may partially clarify the short-term readmissions observed after discharge. Loop diuretics are crucial for alleviating congestion; however, their use often signifies heightened disease severity or diuretic resistance, both of which are indicators of adverse outcomes (47). The observed correlation likely reflects the severity of the underlying condition rather than a direct negative effect of the medication. Thiazide diuretics are infrequently utilized as first-line therapies for congestive heart failure; nevertheless, they could be employed in conjunction with loop diuretics to manage diuretic resistance or regulate refractory volume overload. The frequent use of thiazides typically indicates that patients belong to a cohort with more severe or treatment-resistant conditions, rather than suggesting that thiazides themselves elevate the risk of readmission. Thiazides are frequently administered in conjunction with other medications, such as ACE inhibitors or ARBs, complicating the assessment of their individual effects (48). The observed link between thiazide consumption and readmission likely indicates confounding by indication, where thiazide prescription serves as a clinical marker of advanced or complex heart failure care rather than a direct risk factor.

When considered comprehensively, these findings showed that medication-related predictors of readmission should be analyzed within the context of the clinical situation. Future prospective studies incorporating dosage, treatment sequence, and outpatient follow-up data are necessary to clarify these connections.

#### 4.2.4 Laboratory indications

The results of the meta-analysis showed that LVEF < 40% was a risk factor for unplanned 30-day readmission in patients with CHF. While systolic dysfunction is intuitively associated with negative outcomes, the precise processes by which reduced LVEF results in early readmission remain poorly understood. The variable reporting on functional status, self-care ability, or outpatient follow-up complicates the evaluation of whether LVEF serves as a true physiological driver of readmission or merely as an indicator of disease chronicity and management intensity. Additional study integrating echocardiographic measures with longitudinal clinical data is essential to clarify this link. In addition, although our meta-analysis did not initially include studies evaluating home-based monitoring of BNP or NT-proBNP, existing studies (37–39) have proposed that serial, post-discharge measurement of these biomarkers in the home setting may offer dynamic, real-time information on a patient's hemodynamic status and volume profile. Such an approach could enable earlier identification of subclinical deterioration and prompt therapeutic adjustment by specialists, heart failure nurses, and primary care physicians in a coordinated fashion. BNP or NT-proBNP are well-established prognostic indicators in congestive heart failure, and their serial assessment may more accurately represent disease progression than isolated in-hospital measurements. When incorporated into a systematic follow-up strategy, home BNP/NT-proBNP monitoring could potentially reduce the incidence of unplanned readmissions by facilitating earlier intervention. This method, while not yet often utilized in standard clinical practice, merits additional investigation in forthcoming trials and meta-analyses due to its potential as a low-burden, biomarker-guided follow-up instrument.

#### 4.2.5 Hospitalization-related factors

This study found that shorter LOS or longer LOS were risk factors for 30-day unplanned readmission in patients with CHF. Some studies (24, 49) have shown that shorter LOS may provide insufficient medical care; patients with longer LOS are typically elderly and very sick, as their organ function has deteriorated and they have suffered a physical injury from a severe disease. These circumstances can lead to a worse recovery and a lower body's tolerance to external stimuli, which may cause heart attacks during emergency events and necessitate readmission.

## 5 Strengths and limitations

To our knowledge, this is the first comprehensive systematic review and meta-analysis to assess the global incidence of 30-day unplanned readmission in patients with CHF and synthesize the evidence regarding its risk factors. Using random-effects models, subgroup analyses, and heterogeneity assessments made the pooled estimates stronger by taking into account differences between studies. Stratified analyses based on region, comorbidities, and treatment-related factors enhanced the clinical significance of the findings by pinpointing subgroups with an elevated risk of readmission. This review offers a globally representative perspective on early readmission patterns in CHF by synthesizing evidence from various healthcare systems across continents.

However, this study also had some limitations. First, one significant limitation of this meta analysis is the absence of data from Europe, particularly regarding the various hospital factors (symptoms, signs, clinical scores, and BNP levels) that may affect 30-day readmission rates in CHF patients. European data, which could yield insights into this extensive and varied population, are conspicuously limited in the evaluated studies. This absence could reflect a lack of focus in European healthcare policies on addressing the post-discharge readmission problem, despite its significant economic burden. Second, we limited our analysis to published works in Chinese and English. This criterion may have contributed to some publication bias in the results. Third, due to variations in outcome measures and analytical methods, the available HR and OR data were insufficient to conduct a thorough examination of all risk factors, even in studies with large sample sizes. Moreover, several included studies had small samples (<30 cases), which may reduce the reliability of the pooled estimates and limit confidence in the findings. However, we retained these studies to preserve the completeness of available evidence and because their exclusion did not materially change the pooled results in sensitivity analyses. Lastly, given the insufficient number of studies, the meta analysis and subgroup analysis on the effect size and heterogeneity of some risk factors’ impact could not be conducted.

## 6 Implications for practice and research

This study found several predictors of 30-day unplanned readmission in CHF, and these findings have significant implications for healthcare policy. First, standardized discharge risk assessment, integrating these variables into proven models, could inform the prioritizing of high-risk patients for rigorous follow-up. Policies should guarantee organized early post-discharge communication within 7–14 days through clinic visits or telemedicine to assess stability and compliance (50). Second, multidisciplinary HF teams—consisting of cardiologists, nurses, pharmacists, nutritionists, and primary care physicians can enhance the management of HF and comorbidities, especially in elderly patients or those with CKD or DM (51). Hospital protocols should ensure the beginning and optimization of guideline-directed therapy prior to discharge, with mechanisms for post-discharge titration established. Third,assistance for home-based monitoring, encompassing BNP/NT-proBNP tests, daily weight assessments, and remote blood pressure surveillance, is crucial for patients with diminished LVEF or other comorbidities (52). Such measures could shift CHF care from reactive to preventive, improving outcomes and resource efficiency.

## 7 Conclusions

In our meta-analysis, we found that the incidence of 30-day unplanned readmission for patients with CHF was 17.7%. This finding suggested that age ≥65, COPD, CKD, DM, AF, CHD, cardiomyopathy, NYHA class ≥III or IV, beta-blockers, loop diuretics, thiazides, LVEF < 40%, and shorter LOS and longer LOS were significant independent risk factors for 30-day unplanned readmission in CHF patients. Clinical staff can refer to the results of this study to understand the risk factors associated with the readmission of CHF patients, identify areas of high readmission incidence, and provide early warning to the clinic. This will enable them to take scientific preventive measures to reduce the incidence of readmission among CHF patients.

## Data Availability

The original contributions presented in the study are included in the article/Supplementary Material, further inquiries can be directed to the corresponding author.
