# Supplementary material for: Incidence and influencing factors of 30-day unplanned readmission in chronic heart failure patients: a systematic review and meta-analysis
Source: Front Cardiovasc Med. 2026 Jan 14;12:1663018. doi: 10.3389/fcvm.2025.1663018 (PMC12847312; doi:10.3389/fcvm.2025.1663018)
Supplement: Supplementary file 1 [file Datasheet1.pdf]

Supplementary Appendix S1. Search strategy (Example of PubMed search strategy).

ID	Search strategy
#1	heart failure[MeSH]
#2	heart failure[Title/Abstract] OR cardiac failure[Title/Abstract] OR heart decompensation[Title/Abstract] OR decompensation, heart[Title/Abstract] OR congestive heart failure[Title/Abstract] OR heart failure, congestive[Title/Abstract] OR heart failure, right-sided[Title/Abstract] OR heart failure, right sided[Title/Abstract] OR right-sided heart failure[Title/Abstract] OR right sided heart failure[Title/Abstract] OR heart failure, left-sided[Title/Abstract] OR heart failure, left sided[Title/Abstract] OR left-sided heart failure[Title/Abstract] OR left sided heart failure[Title/Abstract] OR myocardial failure[Title/Abstract]
#3	#1 OR #2
#4	patient readmission*[MeSH]
#5	readmission, patient[Title/Abstract] OR unplanned readmission*[Title/Abstract] OR readmission, unplanned[Title/Abstract] OR rehospitalization*[Title/Abstract] OR hospital readmission*[Title/Abstract] OR readmission*, hospital[Title/Abstract] OR unplanned hospital readmission*[Title/Abstract] OR hospital readmission*, unplanned[Title/Abstract] OR thirty day readmission*[Title/Abstract] OR readmission*, thirty day[Title/Abstract] OR 30 day readmission*[Title/Abstract]
#6	#4 OR #5
#7	risk factors[MeSH]
#8	risk factor*[Title/Abstract] OR factor, risk[Title/Abstract] OR prevalence*[Title/Abstract] OR incidence[Title/Abstract] OR influence factor*[Title/Abstract] OR influencing factor*[Title/Abstract] OR impact factor*[Title/Abstract] OR dangerous factor*[Title/Abstract] OR contributing factor*[Title/Abstract] OR relevant factor*[Title/Abstract] OR relevant factor*[Title/Abstract] OR correlative factor*[Title/Abstract] OR associated factor*[Title/Abstract]
#9	#7 OR #8
#10	#3 AND #6 AND #9

Supplementary Appendix S2.
Risk factors for 30-day unplanned readmissions in patients with CHF

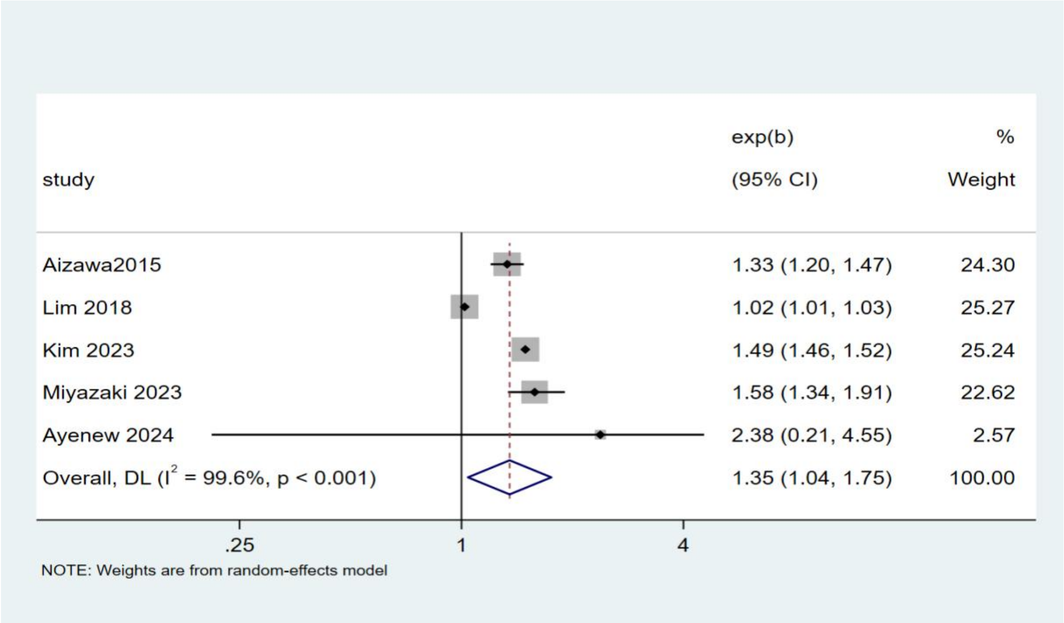

Figure 1. Age

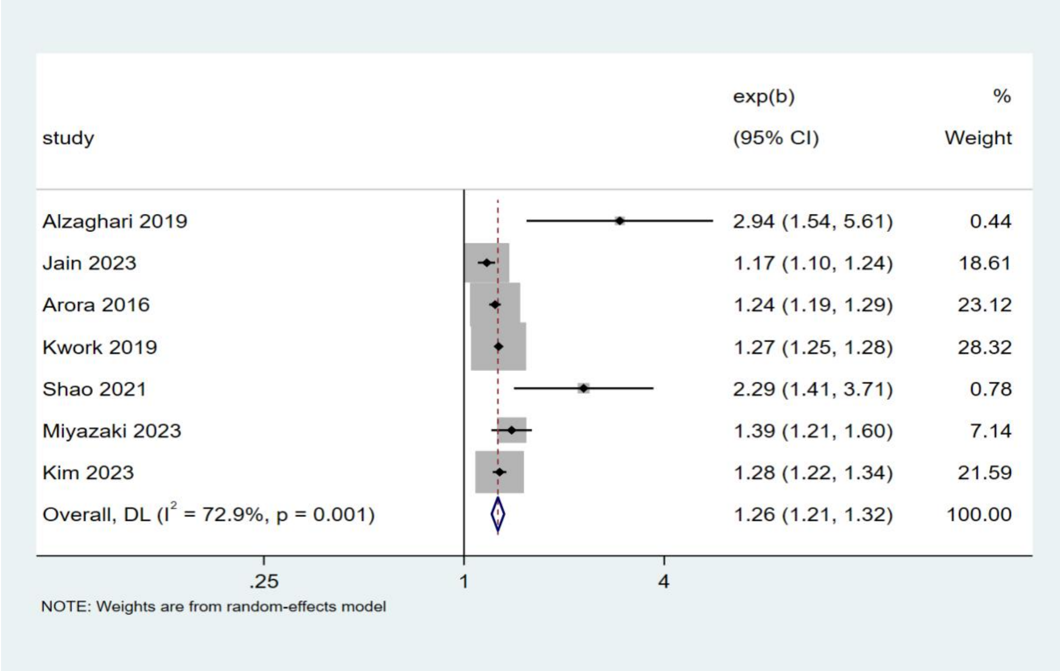

Figure 2. Chronic kidney disease

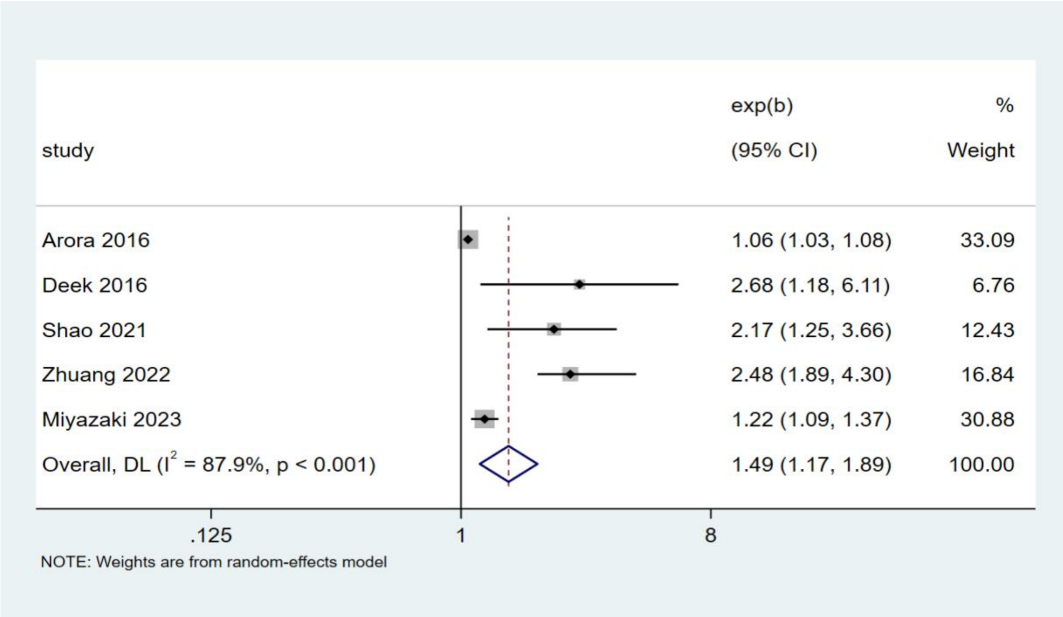

Figure 3. Diabetes mellitus

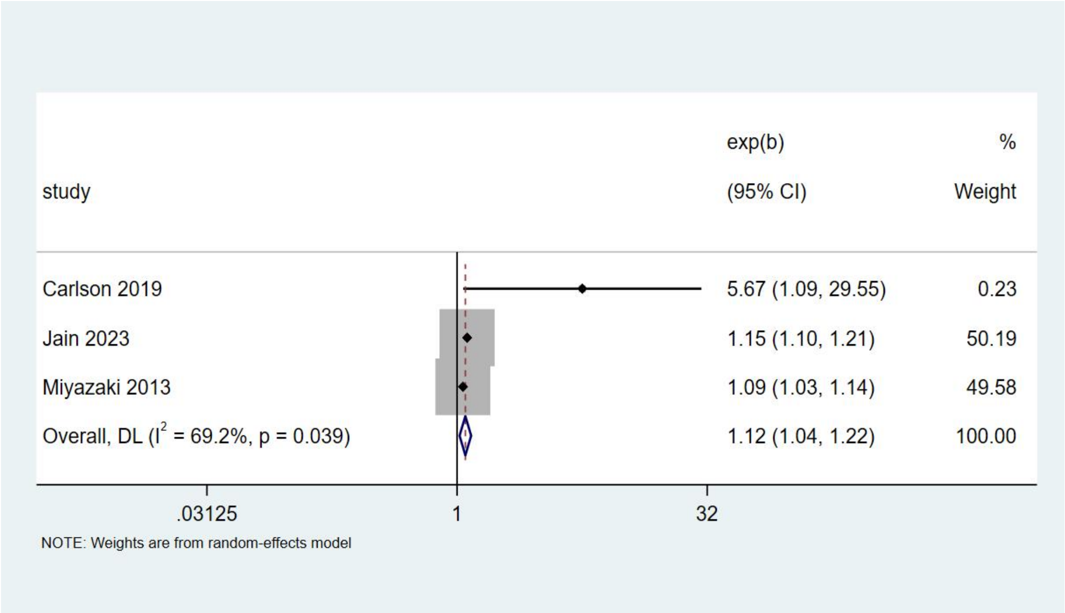

Figure 4. Atrial fibrillation

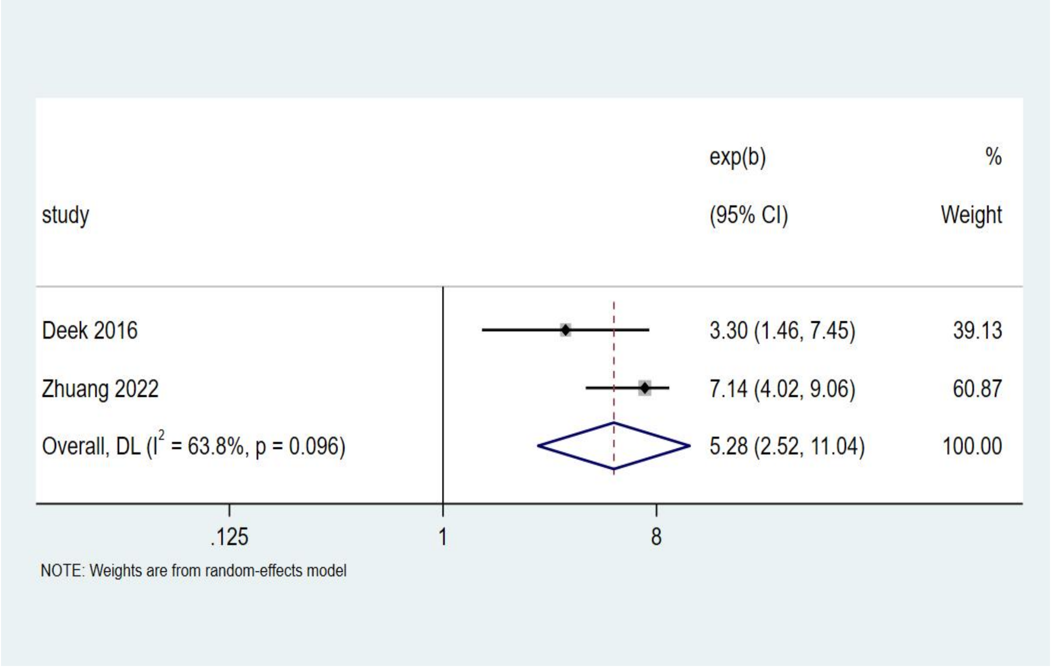

Figure 5. Coronary heart disease

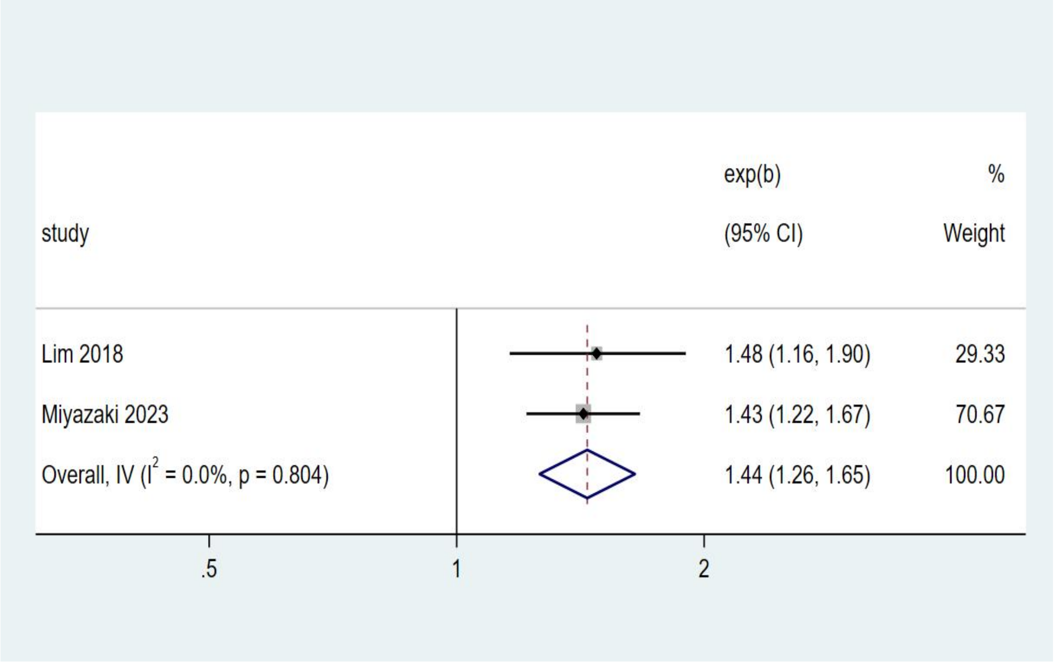

Figure 6. Cardiomyopathy

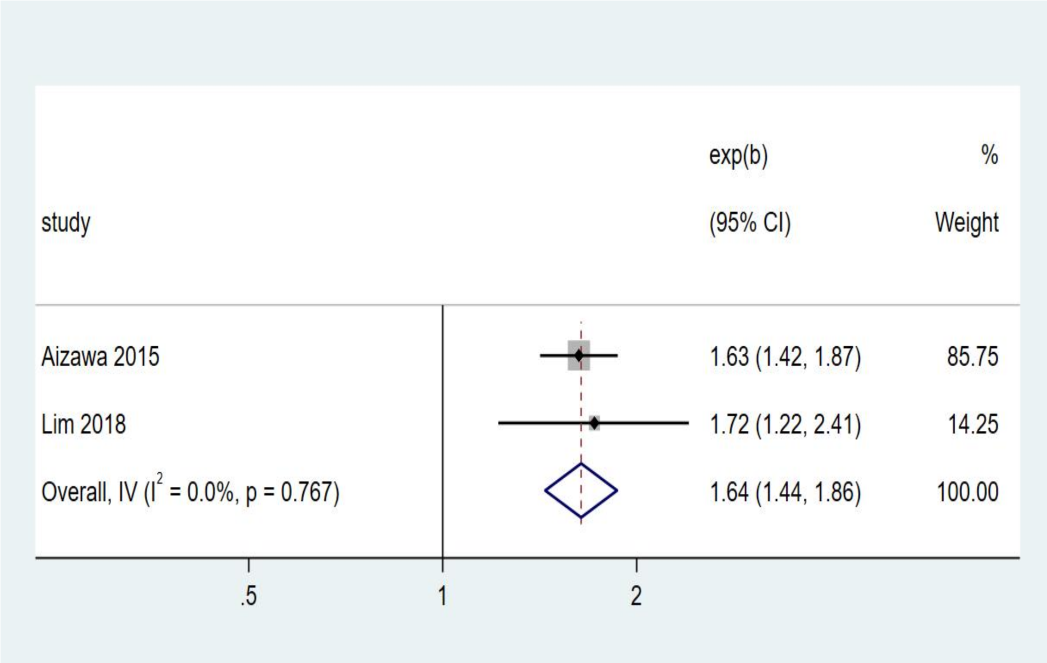

Figure 7. NYHA class≥Ⅲ or Ⅳ

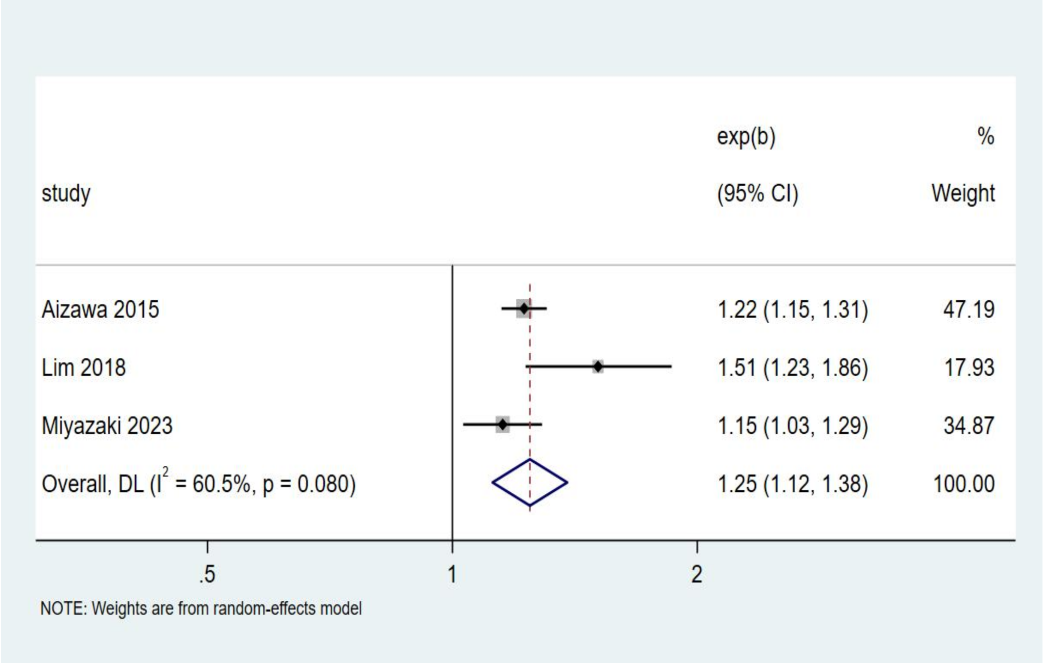

Figure 8. Use of beta-blockers

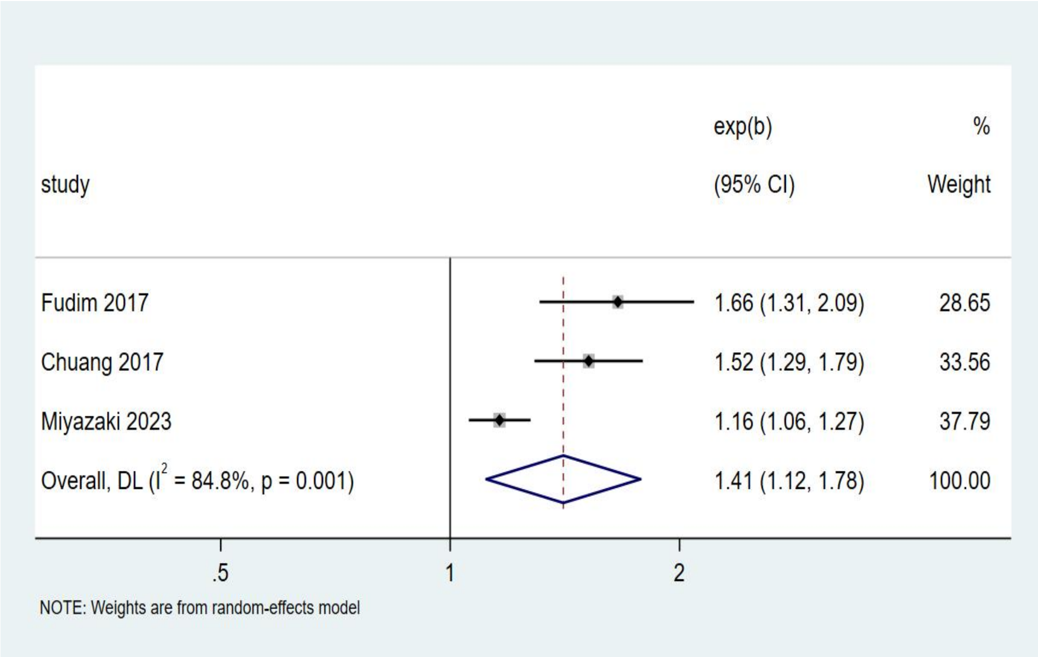

Figure 9. Use of loop diuretics

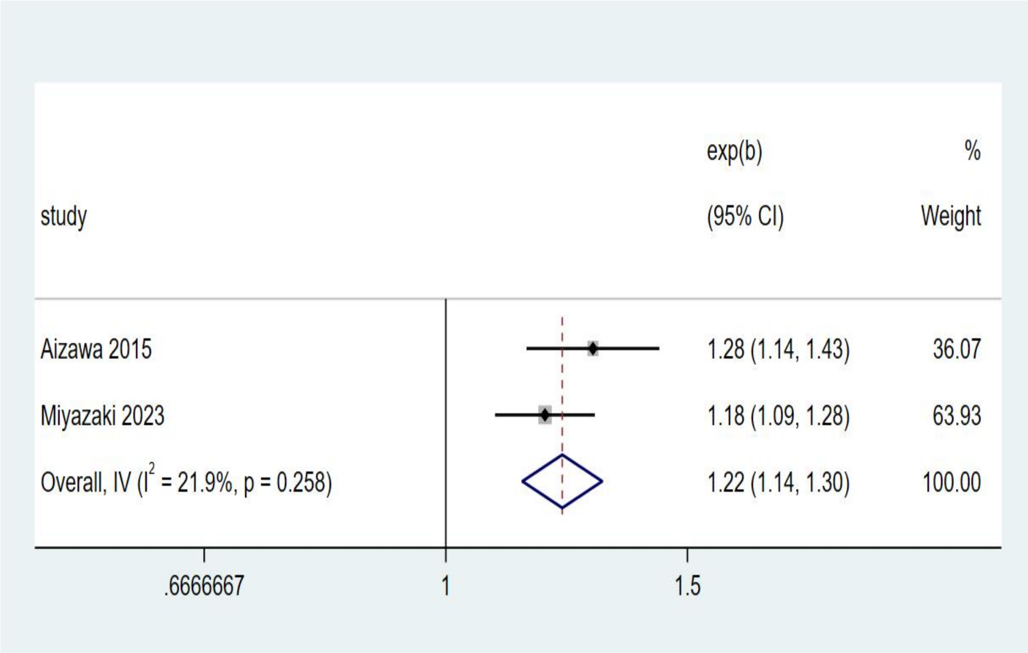

Figure 10. Use of thiazide
